# Breast Cancer Screening Programmes across the WHO European Region: Differences among Countries Based on National Income Level

**DOI:** 10.3390/ijerph14040452

**Published:** 2017-04-23

**Authors:** Emma Altobelli, Leonardo Rapacchietta, Paolo Matteo Angeletti, Luca Barbante, Filippo Valerio Profeta, Roberto Fagnano

**Affiliations:** 1 Department of Life, Health and Environmental Sciences, University of L’Aquila, 67100 L’Aquila, Italy; leonardo.rapacchietta@gmail.com (L.R.); paolomatteoangeletti@gmail.com (P.M.A.); luca.barbante@gmail.com (L.B.); 2 Epidemiology and Biostatistics Unit, Local Health Unit 4, 64100 Teramo, Italy; 3 Department of Community Health, Local Health Unit 4, 64100 Teramo, Italy; valerio.profeta@aslteramo.it; 4 Local Health Unit 4, 64100 Teramo, Italy; roberto.fagnano@aslteramo.it

**Keywords:** breast cancer, screening programme, WHO European Region, income

## Abstract

Breast cancer (BC) is the most frequent tumour affecting women all over the world. In low- and middle-income countries, where its incidence is expected to rise further, BC seems set to become a public health emergency. The aim of the present study is to provide a systematic review of current BC screening programmes in WHO European Region to identify possible patterns. Multiple correspondence analysis was performed to evaluate the association among: measures of occurrence; GNI level; type of BC screening programme; organization of public information and awareness campaigns regarding primary prevention of modifiable risk factors; type of BC screening services; year of screening institution; screening coverage and data quality. A key difference between High Income (HI) and Low and Middle Income (LMI) States, emerging from the present data, is that in the former screening programmes are well organized, with approved screening centres, the presence of mobile units to increase coverage, the offer of screening tests free of charge; the fairly high quality of occurrence data based on high-quality sources, and the adoption of accurate methods to estimate incidence and mortality. In conclusion, the governments of LMI countries should allocate sufficient resources to increase screening participation and they should improve the accuracy of incidence and mortality rates.

## 1. Introduction

Breast cancer (BC) is the most frequent tumour affecting women all over the world, with an incidence rate of 43.1 (per 100,000 ASR-W), a mortality rate of 12.9 (per 100,000 ASR-W), and a 5-year prevalence of 239.9 [1]. In low- and middle-income countries, where its incidence is expected to rise further, BC seems set to become a public health emergency [2], while the highest incidence rates, reported in high-income countries, are partially to be attributed to earlier screening detection [3].

Indeed, in the WHO European Region rates are higher than global rates, incidence being 66.5 (per 100,000 ASR-W) and mortality 16.0 (per 100,000 ASR-W). In EU-28 countries the incidence rate is 80.3 (per 100,000 ASR-W) and the mortality rate 14.4 (per 100.000 ASR-W) [1]. Most EU-28 countries [4], including the UK [5,6,7,8], France [9,10], Italy [11], and Belgium [12,13,14,15], have national cancer prevention population-based (PB) screening programmes not only for BC, but also for cervical cancer (CC) [16] and, as of recently, colorectal cancer (CRC) [17,18]. Within the Council of Europe (CoE), which includes the EU-28 member States (MS) and 19 other countries [18], the right to health is enshrined in the “Right to Protection of Health” [19] and in Article 3 of the Convention on Human Rights and Biomedicine (equal conditions for access to health services) [20,21,22].

In Europe, population-based (PB) mammography screening has reduced mortality by 25%–31%, and by 38%–48% in women receiving adequate follow-up [14]. The level of evidence regarding the usefulness of mammography in reducing mortality in women aged 50 to 74 years is “sufficient” [5]. 

The risk of developing BC is affected by some non-modifiable factors (e.g., age, genetic and familial risk) [23] and by others that can be modified, which are related to lifestyle (e.g., alcohol abuse, tobacco use, and body mass index) [24,25]. Prevention campaigns to reduce the risk attributable to modifiable risk factors should therefore be conducted in all countries.

The aim of the present study is to provide a systematic review of current BC screening programmes in WHO European Region countries to identify possible differences among countries based on gross national income (GNI) [26].

## 2. Materials and Methods

The WHO European area, which is supervised by the WHO EURO office based in Copenhagen (Denmark), includes 53 countries: Albania, Andorra, Armenia, Austria, Azerbaijan, Belarus, Belgium, Bosnia, Bulgaria, Croatia, Cyprus, Czech Republic, Denmark, Estonia, Finland, France, Georgia, Germany, Greece, Hungary, Iceland, Ireland, Israel, Italy, Kazakhstan, Kyrgyzstan, Latvia, Lithuania, Luxembourg, Malta, Monaco, Montenegro, the Netherlands, Norway, Poland, Portugal, the Republic of Moldova, the Russian Federation, Romania, San Marino, Serbia, Slovakia, Slovenia, Spain, Sweden, Switzerland, Tajikistan, the FYR of Macedonia, Turkey, Turkmenistan, Ukraine, the UK, and Uzbekistan. For the purposes of this study, they were grouped according to GNI level referred to per capita Gross National Income (current US$), as indicated by the World Bank [26]: lower-middle income (LMI), $1,026–$4,035; upper-middle income (UMI), $4,036–$12,475; high income (HI), $12,476 or more, and HI OECD countries (Organization for Economic Co-operation and Development), whose average income is $29,016.

### 2.1. Sources of WHO European Epidemiological Data: Search Strategy

The main data source was the GLOBOCAN 2012 website of the International Agency for Research on Cancer (IARC), which provides access to several databases that enable assessing the impact of BC in 184 countries or territories in the world [1,27]. Additional sources were the WHO, IARC, EUCAN and NORDCAN, the European Network of Cancer Registries (ENCR), volume X of the CI5, and the Ministerial and Public Health Agency websites of the individual countries. 

A PubMed search was conducted using Early Cancer Detection OR Cancer Screening OR Screening, Cancer OR Cancer Screening Test OR Early Diagnosis of Cancer OR Cancer Early Diagnosis AND Breast Neoplasm OR Neoplasm, Breast OR Tumours, Breast OR Breast Cancer OR Cancer, Breast OR Mammary Cancer OR Breast Carcinoma AND Europe; Early Cancer Detection OR Cancer Screening OR Screening, Cancer OR Cancer Screening Test OR Early Diagnosis of Cancer OR Cancer Early Diagnosis AND Breast Neoplasm OR Neoplasm, Breast OR Tumours, Breast OR Breast Cancer OR Cancer, Breast OR Mammary Cancer OR Breast Carcinoma AND *“state name”.* Only works published in English in the previous 10 years were considered. A MeSH search was conducted using ((“Breast Neoplasms”[Mesh]) AND “Early Detection of Cancer”[Mesh]) AND Europe; ((“Breast Neoplasms”[Mesh]) AND “Early Detection of Cancer”[Mesh]) AND “state name” for each country. 

The EMBASE database did not provide further relevant results. The registries of some websites and the www.cochranelibrary.com, Scopus, www.clinicaltrials.gov, www.clinicaltrialsregister.eu, Research gate, and Google databases and the national sites of patients’ association were also consulted. All works reporting information considered relevant for the systematic review were examined.

### 2.2. Data Synthesis

**Figure 1:**
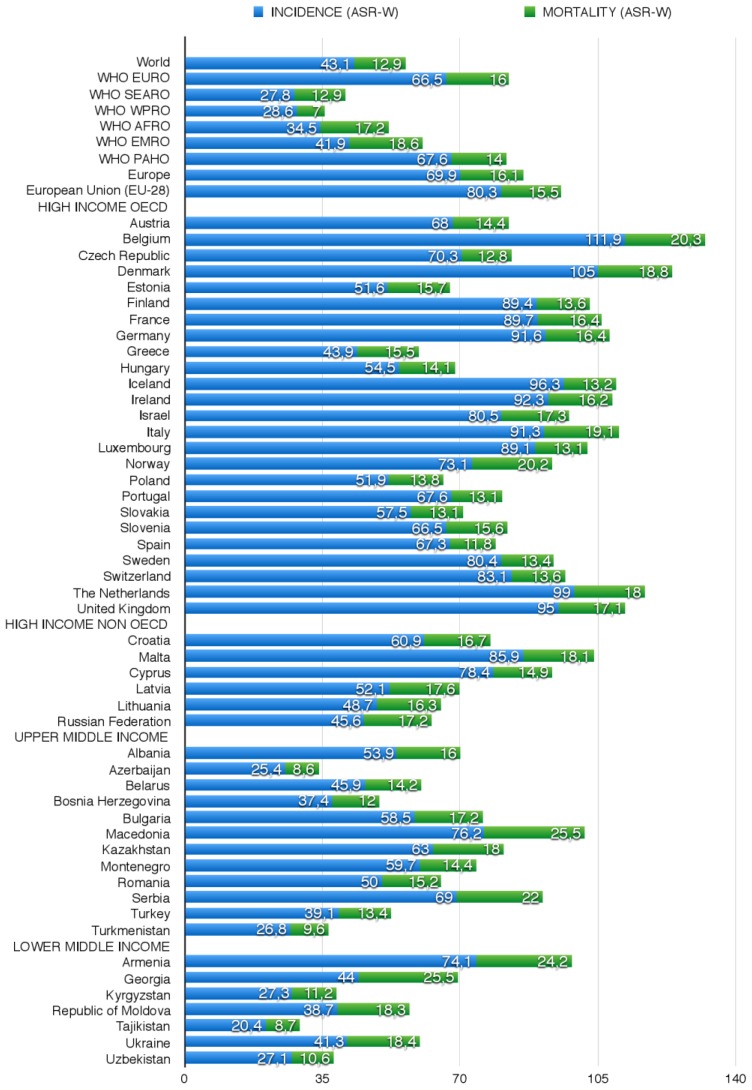
Breast Cancer Incidence and Mortality data and their age standardized rates per 100,000 population (ASR-W), in WHO European Region Countries and in the World, according to GLOBOCAN 2012 (Andorra, Monaco and San Marino not reported).

The 1-, 3-, and 5-year standardized prevalence rates per 100,000 population (ASR-W) for 2012 are reported in Table 1. Incidence and mortality data and their age-standardized rates per 100,000 population (ASR-W) for 2012 are reported in Figure 1. The quality of the epidemiological data of each country, based on Data Sources and Methods according to Mathers [28], is compared in Table 4. The data concerning national primary and secondary prevention campaigns are reported in Table 2. Finally, the information regarding BC screening programmes in the WHO European region is shown in Table 3.

### 2.3. Correspondence Statistical Analysis

Multiple correspondence analysis was performed to evaluate the association among the following variables and identify possible patterns: measures of occurrence (BC incidence, mortality, and prevalence); GNI level (LMI, UMI, and HI); type of BC screening programme in place (national PB/non-national PB; spontaneous/organized) [1,20]; organization of public information and awareness campaigns regarding primary BC prevention (yes/no) of modifiable risk factors (tobacco use, alcohol, obesity, and sedentary lifestyle); type of BC screening services (public health services/public health services + mobile units); year of screening institution (before 2001, 2001 to 2005, after 2005); screening coverage (<50%, 50%–75%, >75%), and data quality. The latter measures included the availability of incidence data, the availability of mortality data, the method adopted to estimate incidence rates, and the method used to estimate mortality rates. As in a previous study by our group [94], these variables were coded as dummy or ordinal variables, as appropriate, and incorporated into the model. Data quality was grouped and defined according to:

1. The availability of incidence data (three categories): “high quality”, from A to C (A = national data or high-quality regional data, coverage > 50%; B = regional data, coverage between 10 and 50%); C = regional data, coverage < 10%); “medium quality”, from D to E (D = national data, rates; E = regional data, rates; and “low quality”, from F to G (F = frequency; G = no data) [28].
2. The availability of mortality data (three categories): “high/medium”, from 1 to 2 (1–2 quality complete vital registration); “low”, 3 to 4 (3 = quality complete vital registration, 4 = incomplete or sample vital registration); and “incomplete or absent”, from 5 to 6 (% = other sources: cancer registries, autopsy, etc; 6 = no data) [28].
3. The quality of the method adopted to estimate incidence rates (three categories): “high” (1). rates projected to 2012 (38 countries); “medium” (from 2 to 4): (2). Most recent rates applied to 2012 population (20 countries), (3). Estimated from national mortality by modelling, using incidence mortality ratios derived from recorded data in country-specific cancer registries (13 countries), (4). Estimated from national mortality estimates by modelling, using incidence mortality ratios derived from recorded data in local cancer registries in neighbouring countries (nine European countries); “low” (from 5 to 9): (5). Estimated from national mortality estimates using modelled survival (32 countries), (6). Estimated as the weighted average of the local rates (16 countries), (7). One cancer registry covering part of a country is used as representative of the country profile (11 countries), (8). Age/sex specific rates for "all cancers" were partitioned using data on relative frequency of different cancers (by age and sex) (12 countries), (9). The rates are those of neighbouring countries or registries in the same area (33 countries) [28].
4. The quality of the method used to estimate mortality rates (three categories): “high” (1). rates projected to 2012 (69 countries); “medium” (from 2 to 4): (2). Most recent rates applied to 2012 population (26 countries), (3). Estimated as the weighted average of regional rates (1 country), (4). Estimated from national incidence estimates by modelling, using country-specific survival (two countries); “low” (from 5 to 6): (5). Estimated from national incidence estimates using modelled survival (83 countries). (6). The rates are those of neighbouring countries or registries in the same area (3 countries) [28].

Finally, incidence, 5-year prevalence, and mortality data were grouped into the following classes, respectively: ≤10/100,000/population, from 10.1 to 20/100,000, from 20.1 to 30/100,000, >30/100,000), ≤100/100,000, 101–150/100,000, 151–200/100,000, 201–250/100,000, >250/100,000), ≤5/100,000, from 5.1 to 10, from 10.1 to 15 and >15/100,000. SAS/STAT software (SAS Institute, Cary, NC, USA) was used for statistical analysis.

## 3. Results

### 3.1. Systematic Review

#### 3.1.1. High-Income OECD Countries

The group of HI OECD countries includes 25 States, 21 EU MS (Austria, Belgium, Czech Republic, Denmark, Estonia, Finland, France, Germany, Greece, Hungary, Ireland, Italy, Luxembourg, the Netherlands, Poland, Portugal, Slovakia, Slovenia, Spain, Sweden, and UK), three CoE MS (Iceland, Norway, Switzerland), and a country with observer status in the CoE (Israel).

The highest BC incidence rates are found in Belgium (111.9), the Netherlands (99), and the UK (95) (vs. 80.3 in EU-28 and 66.5 in the WHO European region) and the lowest in Greece (43.9), Estonia (51.6), Poland (51.9), and Hungary (54.5). Mortality rates are highest in Belgium (20.3), Norway (20.2), Italy (19.1), and Denmark (18.8), and lowest in Spain (11.8), Slovakia (13.1), Portugal (13.1), and Sweden (13.4) (Figure 1).

The 1-year prevalence of BC is > 200 in Denmark and Belgium; its 3-year prevalence is >500 in Denmark, Belgium, the Netherlands, and Finland; and its 5-year prevalence is >800 in Belgium, Denmark, the Netherlands, and Finland. The lowest 1-year and 5-year prevalence rates are found in Greece and Estonia, respectively (Table 1).

In 22 of these 25 countries, data quality is high (A–C) as regards the availability of incidence data, medium/high (1–3) for the mortality data, and medium/high (1–3) for the quality of the method adopted to estimate incidence and mortality rates (Table 4).

Public information and awareness campaigns for primary cancer prevention seem to be more common in the States with a universal health service and in Mediterranean countries (Table 2). Organized BC screening programmes are active in all HI OECD countries except Greece, Czech Republic, Slovakia, and some Swiss cantons, with some differences in the target population (Table 3). In the Czech Republic, a campaign directed at women of screening age who had failed to screen was organized in 2014; nonetheless, screening remains spontaneous, meaning that mammography is prescribed by a specialist (senologist or gynaecologist). In Slovakia and Greece there is no mention of organized screening programmes. In Austria, a national screening programme adopted in 2014 *(Brustkrebs-Früherkennungs programm*) involves rounds at 2-year intervals. Its target population are 45–69 year olds, who are given an e-card offering a mammogram at an approved public or private centre free of charge. Women aged 40–44 years and those aged 70 years or older can also obtain BC screening free of charge, again through activation of an e-card. 

#### 3.1.2. High-Income non-OECD Countries

This group includes nine countries, five EU-28 MS (Croatia, Cyprus, Latvia, Lithuania, and Malta) and four CoE MS (Andorra, Monaco, San Marino, and Russian Federation). BC incidence and mortality rates are highest in Malta (85.9; 18.1); incidence is lowest in the Russian Federation (45.6), and mortality is lowest in Cyprus (14.9) (Figure 1). The highest 1-, 3-, and 5-year prevalence rates are found in Malta and the lowest in the Russian Federation.

Public information and awareness campaigns for primary cancer prevention are carried out in nearly all of these States. All have organized BC screening programmes except the Russian Federation, where screening is spontaneous. 

In five of these nine countries, data quality is high (A-C) as regards the availability of incidence data, medium/high (1–3) for mortality data, and medium/high (1–3) for the quality of the method applied to estimate incidence and mortality (Table 4). Three countries are not evaluable. 

#### 3.1.3. Upper/Middle-Income Countries

This group includes 12 States: Albania (CoE), Azerbaijan (CoE), Belarus, the Federation of Bosnia and Herzegovina (CoE), Bulgaria (EU-28), Kazakhstan, Montenegro (CoE), Romania (EU-28), Republika Srpska (CoE), the FYR of Macedonia (CoE), Turkey (CoE), and Turkmenistan. The FYR of Macedonia has the highest incidence (76.2), mortality (25.5), and prevalence rates as well as 1-, 3-, and 5-year BC prevalence. Incidence and mortality are lowest in Azerbaijan (respectively 25.4 and 8.6), whereas the lowest 1-, 3-, and 5-year prevalence rate is found in Turkmenistan (Figure 1 and Table 1 respectively). 

BC screening is PB and nationwide in Kazakhstan, Serbia, the FYR of Macedonia, and Turkey (Table 3); it is PB but local/regional in Belarus and Bosnia and Herzegovina; and is spontaneous in Albania, Bulgaria, and Romania. There is no evidence of BC screening in Azerbaijan or Turkmenistan (Table 3).

Data quality is high (A–C) as regards the availability of incidence data in three countries; medium/high (1–3) for mortality data in four countries; and medium/high (1–3) for the quality of the method used to estimate incidence in three countries. In all but two countries the quality of the method used to estimate mortality is high (Table 4).

#### 3.1.4. Lower/Middle-Income Countries

This group includes seven countries: Armenia, Georgia, Republic of Moldova, and Ukraine (all CoE MS), Kyrgyzstan, Tajikistan, and Uzbekistan. BC incidence is highest in Armenia (74.1) and mortality in Georgia (25.5); 1-, 3-, and 5-year prevalence peaks in Armenia and is lowest in Tajikistan. PB screening programmes are active in Georgia, Kyrgyzstan, and Uzbekistan; they are also reported in Ukraine in 2002–2006, but they are no longer mentioned. In the other countries there is no evidence of BC screening.

In two of these seven countries data quality is medium/high (A–C) for data source incidence, medium/high (1–3) for data source mortality; the quality of the method used to estimate mortality is medium/high (1–3) (Table 4).

### 3.2. Correspondence Analysis

**Figure 2:**
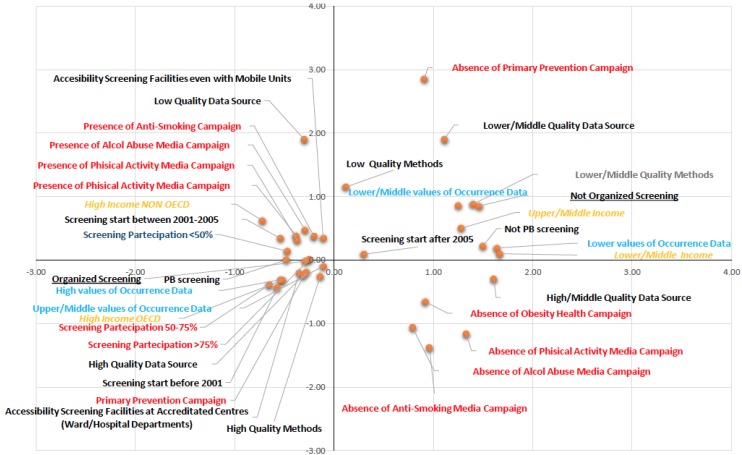
Results of multiple correspondence analysis.

The results of multiple correspondence analysis are represented in Figure 2 (object scores plot). The data provided two dimensions with eigenvalues that explain 65% of the variance: dimension 1 = 0.40 and dimension 2 = 0.25. The first dimension is related to GNI level, year of BC screening institution, type of screening programme in place, and occurrence data; the second dimension relates to the quality of the availability of mortality data, the quality of the method applied to estimate incidence and mortality, and the organization of public information and awareness campaigns for primary prevention of risk factors (tobacco use, alcohol abuse, obesity, and sedentary lifestyle). Multiple correspondence analysis produced clear and interesting patterns, which are represented in the four quadrants of Figure 2. The right upper quadrant is characterized by medium/low GNI, absence of public information and awareness campaigns for primary prevention, low/medium quality of data availability, low quality of the method applied to estimate occurrence rates, low/medium quality of occurrence data, and institution of non-PB organized screening after 2005. The variables found in the left lower quadrant include: HI GNI OECD countries, organized PB screening, 50%–75% and >75% coverage, access to organized PB screening centres, institution of screening programmes before 2001, use of primary prevention public information and awareness campaigns, high/medium-high quality of occurrence data, high quality of the method applied to estimate data, and high quality data availability. The right lower quadrant shows the categories relating to the absence of public information and awareness campaigns for the primary prevention of the risk factors considered in the study (alcohol abuse, tobacco use, obesity, and sedentary lifestyle). Finally, the variables found in the upper left quadrant include HI GNI non-OECD countries, organization of public information and awareness campaigns for the primary prevention of the risk factors considered, institution of screening programmes since 2001–2005, screening coverage <50%, access to approved screening centres, use of mobile units to increase participation, and low-quality data availability.

## 4. Discussion

Over the past three decades, the number of new BC cases has more than doubled worldwide. European incidence and mortality rates vary widely, the highest being found in Belgium (HI; respectively 111.9 and 20.3) and the lowest in Tajikistan (LMI; 20.4 and 8.7). The incidence of BC in developing countries has been increasing by an annual rate of 4.4%. An encouraging finding is that in the countries that have enacted BC screening programmes (all HI States) mortality rates are declining [4]. It has been estimated that 68,000 women aged 15 to 49 years died from BC in LMIs in 2010 as opposed to only 26,000 in HI States [95]. In fact, outcomes in HI countries have improved due to a combination of early screening detection and better treatment [3]. In 1980, 37 women in every 100 new cases died in developing countries; in 2010 the figure was 26 [96]. In contrast, a reduction in the age at BC onset in developing countries is a matter for concern, since these patients account for 44.1% of all cases, while in HI countries BC has become less frequent among women of reproductive age [32]. Mortality would thus appear to correlate inversely with GNI. Mortality rates are a valuable measure of the problem and burden of BC in a country and of the effectiveness of secondary prevention through early detection. Moreover, cancer-specific mortality rates are useful to evaluate the impact of cancer management and treatment. In fact, in developed countries the combination of cancer prevention, early detection, and better treatment has reduced the incidence and mortality of the most common tumours [97,98]. Incidence rates may provide a valuable indicator to investigate risk factors and plan the adoption of prevention programmes. However, their estimation must be accurate if the phenomenon is not to be underestimated, and the absence of a PB or hospital-based cancer registry may be the cause of suboptimal accuracy of data sources. As demonstrated by the data reported above, a very different data quality is found in HI and LMI States, both in terms of the available data sources and of the methods applied to estimate incidence and mortality. This should prompt governments to invest in data source upgrading, to achieve an assessment of the tumour burden as accurate as possible, also with a view to optimising the demand and supply of diagnostic and treatment services. It should also be stressed that high rates of BC detected in advanced phases should prompt the organization of prevention campaigns. 

According to the present study, not all HI countries employ awareness campaigns to prevent important risk factors such as tobacco use and alcohol abuse. HI States lacking them include Austria, Denmark, France, Iceland, and the Netherlands, a UMI country like Bulgaria, and LMI States like Georgia, and Ukraine. The same is true of the prevention of overweight and the promotion of exercise. As regards the enhancement of screening participation, HI States harness multiple means of communication that are sometimes provided in different languages, whereas awareness campaigns in LMI are organized only in Macedonia, Republika Srpska, and Turkey. It is worth stressing that with the exception of Kyrgyzstan, none of the LMI States use mobile units to reach the fraction of the target population who do not respond to the screening invitation. A key difference between HI and LMI States, emerging from the present data, is that in the former screening programmes are well organized, with approved screening centres, the presence of mobile units to increase coverage, the offer of screening tests free of charge; the fairly high quality of occurrence data based on high- quality sources, and the adoption of accurate methods to estimate incidence and mortality, whose accuracy is supported by cancer registries and PB screening. 

## 5. Conclusions

The study suggests the following considerations: first of all, HI Countries like Slovakia, some Swiss cantons, the Russian Federation, and Greece, lack population-based (PB) screening; countries such as Austria, Denmark, France, Iceland, and the Netherlands lack prevention campaigns for the risk factors; countries such as Greece, Hungary, Luxemburg, and Russia lack high-quality data either in terms of data source and of the quality of the method used to estimate incidence and mortality rates. The governments of HI countries should allocate sufficient resources to increase screening participation by harnessing mobile units as well as devising campaigns to enhance women’s awareness of the importance of early BC diagnosis, a goal that would also ensure a more rational utilization of existing approved centres; secondly, they should improve the accuracy of incidence and mortality rates by upgrading the quality of data sources, to avoid being faced with large numbers of BC patients (also) with advanced disease in the near future. High-quality occurrence data are essential to understand cancer trends and devise control strategies. As regards low-middle income countries, they have a less efficient general organization, and the proportion of organized programmes is low in low-income countries while programmes are often absent in middle-income countries. It should however be stressed that for a screening programme to be effective the country should also have suitable facilities to manage all the new cases resulting from early diagnosis, as well as resources to ensure their follow-up. Therefore, small communities lacking specialized medical staff or economic resources to set up screening programmes could rely on nearby centres or regions having the resources and facilities for quality screening. 

## Figures and Tables

**Table 1:** Breast Cancer prevalence for each country of WHO European Region by gross income levels according to World Bank.

High Income OECD Countries	Prevalence Rate *			High Income OECD Countries	Prevalence Rate			Upper Middle Income Countries	Prevalence Rate		
	1 Year	3 Years	5 Years		1 Year	3 Years	5 Years		1 Year	3 Years	5 Years
**Austria**	122.9	348.0	551.4	**Slovakia**	96.4	259.0	388.8	**Albania**	73.7	212.1	338.1
**Belgium**	202.1	571.4	899.4	**Slovenia**	125.2	348.8	540.1	**Azerbaijan**	31.3	83.5	127.0
**Czech Rep**	132.1	360.2	547.2	**Spain**	113.4	325.1	516.2	**Belarus**	76.8	211.5	324.2
**Denmark**	205.0	571.6	887.4	**Sweden**	151.5	434.0	687.4	**Bosnia**	63.2	181.2	287.7
**Estonia**	93.2	254.6	388.3	**Switzerland**	156.6	446.4	705.6	**Kazakhstan**	79.5	210.3	319.0
**Finland**	180.8	513.8	809.2	**The Netherlands**	183.1	521.3	821.4	**FRY of Macedonia**	123.2	354.4	564.5
**France**	168.3	484.1	771.0	**United Kingdom**	174.1	485.2	755.1	**Montenegro**	90.7	260.8	414.7
**Germany**	173.8	488.6	765.7	**High Income non OECD Countries**	**Prevalence rate**			**Romania**	84.2	231.0	353.7
**Greece**	87.4	251.4	400.7		**1 year**	**3 years**	**5 years**	**Serbia**	120.8	344.8	545.9
**Hungary**	98.8	271.2	415.5	**Andorra**	NR	NR	NR	**Turkey**	45.8	122.4	187.0
**Iceland**	158.9	464.5	745.3	**Croatia**	121.7	347.3	549.4	**Turkmenistan**	29.5	78.6	119.9
**Ireland**	145.2	403.9	625.9	**Cyprus**	121.5	347.9	553.0	**Lower Middle Income Countries**	**Prevalence rate**		
**Israel**	123.1	341.6	532.9	**Malta**	156.8	437.4	678.4		**1 year**	**3 years**	**5 years**
**Italy**	169.0	486.5	775.6	**Monaco**	NR	NR	NR	**Armenia**	101.8	270.2	411.2
**Luxembourg**	159.4	456.2	727.4	**Latvia**	96.0	262.7	401.4	**Kyrgyzstan**	28.3	75.4	114.6
**Norway**	131.9	374.2	588.5	**Lithuania**	85.4	234.5	358.6	**Georgia**	63.5	170.0	260.3
**Poland**	92.4	256.3	397.0	**Russia**	78.5	215.0	328.3	**Moldova**	63.7	174.1	265.0
**Portugal**	114.7	324.6	512.2	**San Marino**	NR	NR	NR	**Tajikistan**	18.8	50.4	76.9
								**Ukraine**	69.7	191.0	292.0
								**Uzbekistan**	28.0	74.5	113.3

* 100,000 ASR-W (GLOBOCAN 2012).

**Table 2:** Campaigns of primary prevention and screening promotion in 53 WHO European Countries.

Country	Campaign						
	Control of Cancer Risk Factors				Screening Promotion		Type of BC Screening Services (Public Health Services/Public Health Services + Mobile Units)
	Tobacco	Alcohol	Phisical Activity	Overweight	Media	Languages	
HIGH INCOME COUNTRIES: OECD							
**Austria [29,30]**	NO	NO	NO	YES	Internet; TV; Radio; Brochures; posters	English; Turkish; Bosniac; Croatian; Serbian; Slovenian; Magyar	Accredited centers
**Belgium [12,13,14,15]**	YES	YES	YES	YES	Internet; Brochures; Posters	French; Netherlands	Accredited centers
**Czech Rep [31,32,33]**	YES	YES	YES	YES	Internet;	Česky; English	Accredited centers
**Denmark [34,35,36,37]**	NO	NO	YES	YES	Internet	Danes; English; Turkish; Somali; Bosnian; Arabic; Farsi; Urdu; Kalaallisut	Accredited centers
**Estonia [4,38]**	YES	YES	YES	YES	Internet	Estonia; English	Accredited centers, mobile mammography units
**Finland [4,39]**	YES	YES	YES	YES	Internet	Finnish; Swedish; English	Accredited centers
**France [9,10]**	NO	NO	YES	YES	Internet; TV; Radio; Brochures; Posters	French, English	Accredited centers
**Germany[40,41]**	YES	YES	YES	YES	Internet	German; English	Accredited centers
**Greece [42]**	YES	YES	YES	NO	Internet	Greek; English	Accredited centers
**Hungary [43,44,45]**	YES	NO	NO	NO	Internet	Turkish	Accredited centers
**Iceland [46]**	NO	NO	NO	NO	Internet	Icelandic; English; Polish	Accredited centers
**Ireland [47]**	YES	YES	YES	YES	Internet, Smartphones app	English, Irish	Accredited centers, mobile mammography units
**Israel [48]**	YES	NO	YES	YES	Internet	Israeli; Arabic, English	Accredited centers, mobile mammography units
**Italy [11,49,50]**	YES	YES	YES	YES	Internet; TV; Radio; Brochures	Italian	Accredited centers, mobile mammography units
**Luxembourg [51,52,53]**	YES	NO	YES	YES	Internet; Brochures	French, German	Accredited centers
**Norway [54,55,56,57]**	YES	YES	YES	NO	Internet; Brochures	Norwegian; English	Accredited centers
**Poland [4,58,59]**	YES	YES	YES	NO	Internet	Polish (mi sembra che lo screening sia iniziato di recente. Non ho trovato un sito ufficiale …)	Accredited centers
**Portugal [60,61,62]**	YES	YES	YES	NO	NR	Portuguese	Accredited centers, mobile mammography units
**Slovakia [4]**	YES	YES	YES	YES	NR	NR	NR
**Slovenia [4,63]**	YES	YES	YES	NO	Internet	Slovenian; English	Accredited centers; mobile mammography units
**Spain [64,65]**	YES	YES	YES	YES	Internet	Spanish	Accredited centers mammography centers
**Sweden [66]**	YES	YES	YES	YES	Internet	Swedish; English	Accredited centers
**Switzerland [67,68]**	YES	YES	YES	YES	Internet; TV; Radio, Brochures;	English; Turkish; Bosniac; Croatian; Serbian; German; French; Italian; Spanish; Portuguese; Albanian	Accredited centers
**The Netherlands [56,57,58,59]**	NO	NO	NO	YES	Internet	Nederlands; English; Turkish; Arabic	Accredited centers; mobile mammography units
**United Kingdom [5,6,7,8]**	YES	YES	YES	YES	Internet	English	Accredited centers
**HIGH INCOME NON OECD**							
**Andorra [69]**	YES	YES	YES	YES	Internet	Catalan; Spanish; French; Portuguese; English	Accredited centers
**Croatia [70]**	YES	YES	YES	NO	Internet; Brochures	Croatian	Accredited centers
**Cyprus [71,72,73]**	YES	YES	NR	NR	NR	NR	Accredited centers
**Malta [4,74]**	YES	YES	YES	YES	Internet	English	Accredited centers
**Monaco [75]**	YES	YES	YES	YES	Internet; TV; Radio; Brochures; Posters	French; English	Accredited centers
**Latvia [4]**	YES	YES	YES	NO	Internet	Latvian	Accredited centers
**Lithuania [4]**	YES	YES	YES	YES	Internet	Lithuanian; English	NR
**Russian Fed [76]**	YES	YES	YES	YES	NR	NR	NR
**San Marino [77]**	YES	YES	YES	YES	Internet	Italian	Accredited centers
**UPPER MIDDLE INCOME**							
**Albania [78]**	NO	YES	NO	NO	NR	NR	Accredited centers
**Azerbaijan**	NO	NO	NO	NO	NR	NR	NR
**Belarus [79]**	NR	NR	NR	NR	NR	NR	NR
**Bosnia [80]**	NR	NR	NR	NR	Unrealised	Unrealised	Unrealised
**Bulgaria [4]**	NO	NO	NO	NO	Internet	Italiano	Accredited centers
**Kazakhstan [81]**	NR	NR	NR	NR	NR	NR	NR
**FRY of Macedonia [82]**	YES	YES	YES	YES	Internet, Stampa	Macedonian	Accredited centers
**Montenegro [83,84]**	YES	YES	YES	YES	NR	NR	NR
**Romania [4]**	YES	NO	NO	NO	NR	NR	NR
**Serbia [85,86]**	YES	NO	NO	NO	Internet	Serbian; English	Accredited centers
**Turkey [87]**	YES	NO	YES	YES	Internet	Turkish; English	Accredited centers
**Turkmenistan**	YES	YES	YES	YES	NR	NR	NR
**LOWER MIDDLE INCOME**							
**Armenia**	YES	YES	YES	YES	NR	NR	NR
**Georgia [88]**	NO	NO	NO	NO	Internet	Georgian; English	Accredited centers
**Kyrgyzstan [89]**	YES	YES	YES	YES	Brochures; Conferences; seminars	Kyrgyz; Russian	Accredited centers; mobile mammography units
**Moldova**	YES	YES	NO	NO	NR	NR	NR
**Tajikistan**	YES	YES	YES	YES	NR	NR	NR
**Ukraine [90]**	NO	NO	NO	NO	NR	NR	NR
**Uzbekistan [91]**	NO	NO	NO	NO	Internet	Uzbek	NR

Health Expenditure: data available from World Bank website. Referred to 2014; Cancer policy: data available from WHO web site. All data are referred to a survey (2014); NR: not reported.

**Table 3:** Distribution of Breast Cancer screening programmes in 53 WHO European Countries as of July 2016.

Country	EURO Area	Type	Regions	Start Program	Natw Coverage	Test	Age Target	Views	Double Reading	Screening Interval	Recall %	Level of Participation %	Payment Policy
**HIGH income: OECD countries**													
Austria [35,36]	EU28	PB	All nation	2014	-	DM,US	45–69	2	Yes	2	-	-	
		NPB	All nation	2014	-	DM, US	40–44; >70	2	-	-	-	-	Free of charge
			Tyrol (Innsbruck and hinterland)	2007–2008	-	DM, US	40–59	2	No	1 year 40–59 2 years 60–69	3.1	55.5	Free of charge
Belgium [12,13,14,15]	EU28	PB	Wallonie-Bruxelles	2000	-	DM, US	50–69	2	Yes: if necessary 3	2	-	-	Free of charge
		PB	Flanders	2001	-	DM, US	50–69		-	2	-	32.7	-
Czech Rep [36,37,38]	EU28	NPB	All nation	2002	2007	MM	45–69	2	Yes	2	-	70.0	NA
		PB	All nation	Jan–Dec 2014		MM	45–70	2	Yes	2	-	-	
Denmark [39,40,41,42]	EU28		All nation	2001	2008–2010	DM	50–69	2	Yes	2	Initial: 4.3 Later: 1.8	73.0 *	Free of charge
Estonia [4,43]	EU28		All nation	2002	2007	DM	50–65	2	Yes	2	3.1	53.0	Free of charge
Finland [4,44]	EU28	PB	All Nation	1987	1992	DM,US	50–69	2	Yes	2	2.7	84.0	Free of charge
France [9,10]	EU28	PB	All nation	1989	2004	MM,DM,CBE	50–74	2	Yes	2	1.3	52.7	Free of charge
Germany [45,46]	EU28	PB	All Nation	2002	2009	DM	50–69	2	Yes	2	North Westphalia (2005–2009) Initial: 6.1 Subsequent: 3.4	54.1	Free of charge
Greece [47]	EU28	NPB	Pilot	2004–2009	-	MM	40–69	2	-	1-2	-	-	
Hungary [48,49,50]	EU28	PB	All nation	1995 (PILOT)	2002	DM	45–65	2	Yes	2	7.2	56.3	Free of charge
Iceland [52]	EU19	PB	All nation	1987	1989	DM	40–69	2	Yes	2	4.1	62.0	Free of charge
Ireland [53]	EU28	PB	All nation	2000	2007	DM	50–64	2	Yes	2	Initial: 8.4 Subsequent: 2.8	74.2	Free of charge
Israel [54]	EU19	PB	All nation	1997	2005	MM,DM	50–74	-	-	2	-	72.0	Free of charge
Italy [11,54,55]	EU28	PB	All nation	1990		DM,US	50–69	2	Yes	2	5.4	North: 61.0 Centrale: 56.0 South and Islands: 40.0	
			Emilia Romagna	2010		DM	45–74	2	Yes	45–49 (1 yr) 50–74 (2 yrs)			Free of charge
		NPB	Piedmont	2006		DM	45–49 70–75	2	Yes	1 year2 years			Free of charge
		PB	Lombardy	2012						2 years		70.0	Free of charge
Luxembourg [56,57,58]	EU28	PB	All nation	1992	1992	DM	50–69	2	Yes	2	5.4	64.0	Free of charge
Norway [59,60,61,62]	EU19	PB	All nation	1995	2005	DM	50–69	2	Yes	2	Initial: 46.0 Subsequent: 2.6	76.0	
Poland [4,63,64]	EU28	PB	All nation	2006	2007	MM,DM	50–69	2	Yes	2	2.4	40.0	Free of charge
Portugal [65,66,67]	EU28	PB	All nation			DM	45–69	2	Yes	2		60.0	
			Region Centro	1990	2014							63.0	Free of charge
			Lisboa and Vale do Vale do Tejo	1991								50.0	
			Alentejo	1997	2014							66.0	
			Algarve	2005	2014							66.0	
			Region Norte	2009								58.0	
Slovakia [4]	EU28	NPB	-	-	-	-	40+	-	-	2	-	-	Free of charge
Slovenia [4,68]	EU28	PB	All nation	2008	-	DM	50–69	2	Yes	2	Initial: 4.8 Subsequent: 2.3	77.3	
Spain [69,70]	EU28	PB	Andalucia, Castile-La Mancha, Valencian Community, Navarra La Rioja, City of Ceuta, City of Melilla	1990–2001	1992–2005	DM	45–69	2	Yes	2		67.0	Free of charge
		PB	Aragon, Asturias, Balearic Islands, Cantabria, Castile-Leon, Catalonia, Extremadura, Galicia, Madrid, Murcia, Basque Country	1991–1998	1996–2009	DM	50–69	2	Yes	2			
Sweden [71]	EU28	PB	Sakaraborg, Stockholm Kronoberg Vrmland Vasterbotten Jamtland		1997	MM,DM	50–69	2	Yes	2–1.5	-	72.0–91.0	Free of charge
			Dalarna	1977			40–70					88.0	
			Vastmanland, Gotland	1986			40–69					87.0 89.0	
			Malmo	1977			46–69					66.0	
			Angelholm, Kristianstand, Bohus Halland	1986–1989			50–74					70.0–90.0	
			Gavelborg, Ostergotland, Kalmar, Jonkoping, Malmohus, Alvsborg North, Alvborg South g, Orebro, Uppsala, Sodermanland, norbotten Vasternorrland	1974–1989			40–74					80.0–86.0	
Switzerland [72,73]	EU19	PB	Basilea, Berna, Friburgo, Ginevra, Giura, Grigioni-Neuchatel, San Gallo, Turgovia, Vaud, Vallese	1999	1999	MM,DM	50–70	2	Yes	2	N/A	48.2	Free of charge
		NPB	Other cantons										
The Netherlands [92]	EU28	PB	All nation	1989	1997	MM, DM	50–75	2(1)	Yes	2	-	80.0	Free of charge
United Kingdom [5,6,7,8]	EU28	PB	All nation	1988, 2004 (ext 50–70)	1995	DM	50–70	2	-	3	Initial: 7.4 Subsequent: 3.6	76.0	Free of charge
			Northern Ireland	1990			50–70						
			Scotland				50–70						
			Wales	1989			50–70						
**HIGH INCOME NON OECD COUNTRIES**													
Andorra [75]		PB	All nation	Na		MA	50–69	NA	NA	2	Na	Na	Free of charge
Croatia [76]	EU28	PB		Oct 2006		DM	50–69	2	Yes	2 years		60.0	
Cyprus [77,78,79]	EU28	PB	All nation	2003	2007	DM	50–69	2	-	2	-	50.0	
Malta [4,80]	EU28	PB	All nation	2007	2009	DM	50–60	2	2 (+1)	3	17.1	58.1	Free of charge
Monaco [81,82]		PB	All nation	1994		DM, US	50–80	2	-	2	-	-	
Latvia [4]	EU28	PB	All nation	2008	2009	DM, US	50–69	2		2	N/A	34.2	Free of charge
Lithuania [4]	EU28	PB	All nation	2005	-	DM	50–69	2		2			
Russian Fed [83]	EU19	NPB	Khanty-Mansiysky autonomous Region Yugra	2007–2012		DM	>40	2	No	2		67.5	
San Marino [84]	EU19	PB	All nation	1993	1993	DM,US	35–74	2	N/A	2	N/A	76.0	Free of charge
**UPPER MIDDLE INCOME COUNTRIES**													
Albania [85]	EU 19	NPB	Tirana	2007–2008									-
Azerbaijan	EU 19	N/A	N/A	N/A	N/A	N/A	N/A	N/A	N/A	N/A	N/A	N/A	N/A
Belarus [86]	OEI	NPB	N/A	N/A	N/A	N/A	N/A	N/A	N/A	N/A	N/A	N/A	N/A
Bosnia and Herzegovina [4,87]	EU 19	PB	Sarajevo	2000–2006		M	45–55	-	N/A	N/A	N/A	53.5	Free of charge
Bulgaria [4]	EU28	NPB	All nation	2000		FM	45–69	N/A	N/A	N/A	N/A	N/A	N/A
Kazakhstan [88]	OEI	PB	All nation	2008		DM		N/A	Yes	2			N/A
FRY of Macedonia [89]	EU 19	PB	All nation	2007		M ,US	50–69	2	N/A	2	N/A	N/A	N/A
Montenegro [90]	EU 19	PB	Podgorica, Danilovgrad, Cetinje and Kolašin.			DM	50–6940–69		Yes	2	N/A	70%	Free of charge
Romania [4]	EU28	NPB	All nation	N/A	N/A	N/A	N/A	N/A	N/A	N/A	N/A	N/A	Free of charge
Serbia [91,92]	EU 19	PB	All nation	2013	2014	M	50–69	-	Yes	2	-	75.0	Free of charge
Republika Srpska		N/A	N/A	N/A	N/A	N/A	N/A	N/A	N/A	N/A	N/A	N/A	N/A
Turkey [93,94]	EU 19	PB	All nation	2009	2009	DM, US	50–69	2	Yes	2	N/A	20.0	N/A
Turkmenistan	OEI	N/A	N/A	N/A	N/A	N/A	N/A	N/A	N/A	N/A	N/A	N/A	N/A
**LOWER MIDDLE INCOME COUNTRIES**													
Armenia	EU 19	N/A	N/A	N/A	N/A	N/A	N/A	N/A	N/A	N/A	N/A	N/A	N/A
Georgia [95]	EU 19	PB	All nation	NA		MA	40–70		2	2	YES	75.0	Free of charge
Kyrgyzstan [96]	EU 19	PB	All nation	2007	2007	DM	40–69	-	-	3	-	-	
Republic of Moldova	EU 19	N/A	N/A	N/A	N/A	N/A	N/A	N/A	N/A	N/A	N/A	N/A	N/A
Tajikistan	OEI	N/A	N/A	N/A	N/A	N/A	N/A	N/A	N/A	N/A	N/A	N/A	N/A
Ukraine [97]	EU 19	N/A	All nation	2002–2006	N/A	N/A	N/A	N/A	N/A	N/A	N/A	N/A	N/A
Uzbekistan [93]	OEI	PB	All nation	2009	2013	N/A	N/A	N/A	N/A	N/A	N/A	N/A	N/A

DM: Digital Mammografy, MA: Mammography Us: Ultrasounds; EU 28: Country of European Union EU 19: Country of European Council outside of EU 28 OEI: outside of European institutions; N/A: not available; PB: population-based; NPB: non-population-based.

**Table 4:** Epidemiological data quality for the 53 WHO European area nations.

	Quality of Data				
Country	Data Source			Methods	
	Incidence	Mortality	Cancer Registry *	Incidence (a)	Mortality (b)
***HIGH INCOME OECD COUNTRIES***					
Austria	A	2	Austria, Tyrol, Vorarlberg	1	1
Belgium	A	2	National	2	2
Czech Rep	A	2	National	1	1
Denmark	A	2	National	1	1
Estonia	A	1	National	1	1
Finland	A	1	National	1	1
France (metropolitan)	B	2	Bas−Rhin, Calvados, Doubs, Haut−Rhin, Hérault, Isère, Loire−Atlantique, Manche Somme, Tarn, Vendée	3	1
Greece	G	3	-	4	1
Germany	B	2	Brandenburg, Bremen, Free State of Saxony, Hamburg, Mecklenburg−Western Pomerania, Munich, North Rhine−Westphalia; Saarland, Schleswig−Holstein	1	1
Hungary	G	1	-	4	1
Iceland	A	1	National	1	1
Ireland	A	1	National	1	1
Israel	A	2	National	1	1
Italy	B	2	Biella, Brescia, Catania and Messina, Catanzaro, Como, Ferrara, Florence and Prato, Friuli-Venezia Giulia, Genoa, Latina, Lecco, Lombardy South, Mantua, Milan, Modena, Naples, Nuoro, Palermo, Parma, Ragusa, Reggio Emilia, Romagna, Salerno, Sassari, Sondrio, South Tyrol, Syracuse, Trapani, Trento, Turin, Umbria, Varese, Veneto	3	1
Luxembourg	D	2	-	4	1
Netherlands	A	2	National, Eindhoven	1	1
Norway	A	2	National	1	1
Poland	C	3	Cracow, Kielch, Lower Sileisa, Podkarpackie	3	1
Portugal	C	3	Azores	4	1
Slovak Rep	A	1	National	1	1
Slovenia	A	1	National	1	1
Spain	B	2	Albacete, Asturias, Basque Country, Canary Islands, Ciudad Real Cuenca, Girona, Granada, La Rioja, Mallorca, Murcia, Navarra, Tarragona	3	1
Sweden	A	2	National	3	1
Switzerland	B	2	Basel, Geneva, Graubünden and Glarus, Neuchâtel, St Gall−Appenzell, Ticino, Valais, Vaud, Zurich	3	1
UK	A	1	England, East of England Region; North Western, Northern and Yorkshire, Oxford Region; England, South and Western Regions, Thames, Trent West Midlands, Northern Ireland; Scotland Wales	1	1
***HIGH INCOME NON OECD COUNTRIES***					
Andorra	-	-	Hospital based (National)	-	-
Croatia	A	2	National	1	1
Cyprus	A	3	National	2	2
Malta	A	1	National	1	1
Monaco	-	-	Hospital based (National)	-	-
Latvia	A	1	National	1	1
Lithuania	A	1	National	1	1
Russian Fed	D	2	Saint Pethersburg	1	1
San Marino	-	-	National (Activeted 2013)	-	-
***UPPER MIDDLE INCOME COUNTRIES***					
Albania	G	3	Tirana and 36 districts	4	1
Azerbaijan	G	2	Activated in 2015	5	2
Belarus	A	2	National	1	2
Bosnia	D	5	-	2	2
Bulgaria	A	2	National	1	1
Kazakhstan	G	2	National	5	2
Macedonia	G	3	National	4	1
Montenegro	G	6	-	9	6
Romania	E	1	Timisoara, Cluj	4	1
Serbia	B	2	Subnational (Serbia, Central)	4	1
Turkey	C	6	Antalya, Edirne, Izmir, Trabzon	6	5
Turkmenistan	G	2	-	5	1
***LOWER MIDDLE INCOME COUNTRIES***					
Armenia	G	3	-	5	2
Kyrgyzstan	G	2	-	5	1
Georgia	G	2	-	5	2
Moldova	A	2	-	1	1
Tajikistan	G	3	-	5	2
Ukraine	A	2	National	2	2
Uzbekistan	G	2	-	5	2

* Cancer registry according to IARC.
